# The V. S. and Public Health in Italy

**Published:** 1901-12

**Authors:** 


### THE V. S. AND PUBLIC HEALTH IN ITALY.

The University of Rome has reorganized a long-felt want and instituted a diploma of public health for veterinary graduates on similar lines to the medical D.P.H. The subjects for the D.V.H. are:

1. Bacteriology, specially as applied to diagnostic injections, immunization, and curative means.
2. Contagious diseases, including practical recognition.
3. "Zootecnia" of meat and milk products; hygiene of the dairy and cow sheds, sale, slaughter, and export of meat producers.
4. Chemistry and microscopy of food products derived from animals.
5. Practical demonstrations on the inspection of live and dead meat producers.
6. Administration of legislative, statistical, and police sanitary methods affecting animals.—*La Clinica Vet.*

